# Racism and Older Black Americans’ Health: a Systematic Review

**DOI:** 10.1007/s11524-021-00591-6

**Published:** 2022-01-07

**Authors:** S. E. LaFave, J. J. Suen, Q. Seau, A. Bergman, M. C. Fisher, R. J. Thorpe, S. L. Szanton

**Affiliations:** 1 Johns Hopkins University School of Nursing, Baltimore, MD USA; 2 Johns Hopkins Bloomberg School of Public Health, Baltimore, MD USA; 3 Johns Hopkins University School of Medicine, Baltimore, MD USA

**Keywords:** Racism, Older adults, Systematic review, Discrimination, Social determinants of health

## Abstract

We reviewed research that examines racism as an independent variable and one or more health outcomes as dependent variables in Black American adults aged 50 years and older in the USA. Of the 43 studies we reviewed, most measured perceived interpersonal racism, perceived institutional racism, or residential segregation. The only two measures of structural racism were birth and residence in a “Jim Crow state.” Fourteen studies found associations between racism and mental health outcomes, five with cardiovascular outcomes, seven with cognition, two with physical function, two with telomere length, and five with general health/other health outcomes. Ten studies found no significant associations in older Black adults. All but six of the studies were cross-sectional. Research to understand the extent of structural and multilevel racism as a social determinant of health and the impact on older adults specifically is needed. Improved measurement tools could help address this gap in science.

## Introduction

Older Black Americans experience earlier mortality and higher rates of many chronic conditions compared to their White counterparts. [1] Since race is a social construct (or, as Dr. Camara Jones defines, a “social interpretation of how one looks”), it is inherently not a risk factor for disease. Race is instead a risk factor for racism which is associated with negative health outcomes. [2–4] A Black American aged 65 years today lived the first decade of their life during legally enforced racial segregation during the Jim Crow Era. The current generation of older Black Americans experienced both legalized discrimination prior to 1965, as well as less blatant but dangerously persistent systems of racism that continues to impact Americans of Color today. [5] Understanding and addressing the influence of racism on health is crucial to targeting health disparities in older adults, as well as in reducing overall morbidity and mortality from chronic health conditions in this country.

Racism can be classified into three different levels of operation: interpersonal, institutional, and structural. Interpersonal racism affects health at the individual level through encountering discrimination based on race in everyday social interactions. [6] This individual level of racism includes microaggressions and unfair treatment that can be harmful regardless of whether there were malicious intentions. Institutional racism affects health when agencies or organizations in a particular sector discriminate against people based on their race. [5] If a company underpays employees of Color, or if hospitals provide different qualities of care to patients based on their race, institutional racism invariably widens disparities. Similarly, this occurs even if inequitable policies and subsequent practices are neither intentional nor obvious. Finally, structural racism is enacted through discrimination that occurs across institutions, systems, and contexts through coinciding racist policies and practices that do not depend on the decisions of just one individual, organization, or sector. [5] These overlapping policies and practices preferentially offer opportunities, advantages, services, or supports to one racial group while depriving other racial groups of those same benefits. [5, 7] For example, a person could accumulate exposure to structural racism due to the systematic denial of home loans combined with the disproportionate incarceration rates experienced by Black Americans. [8, 9] Americans of Color are impacted by the coinciding effects of racism inflicted at the interpersonal, institutional, and structural levels. [5, 6]

Racism is a fundamental cause of health disparities in the USA, but the specifics of how and to what extent racism affects health outcomes are not well-established. Racism affects every age group of every minoritized racial and ethnic group in the USA. It is important to study racism and health in older Black Americans because of the uniquely harmful effects that the Jim Crow Era had in this group throughout history and because of the magnitude of health disparities between older Black and older White Americans. Additionally, a focus on older adults allows for improved understanding of cumulative disadvantage over the life course – or the increasing effects of inequality on health over time. [10]

Researchers have conducted systematic reviews specific to racism or discrimination and health. [11–16] Previous reviews have examined the relationship between racism or discrimination and one type of health outcome such as hypertension, [13, 17] allostatic load, [18] or mental health. [12, 14] Other reviews focused on a specific level of racism, such as perceived interpersonal racism. [11] To our knowledge, no reviews have examined the relationship between all levels of racism and all health outcomes in older Black Americans. In this study, we aim to systematically review research examining the relationship between racism at the interpersonal, institutional, and structural levels and any health outcome in older Black Americans. Our objective is to provide a comprehensive assessment of the strengths and gaps of the current body of scholarly literature on the health impacts of racism in older Black Americans to inform future research on this important and understudied contributor to health disparities.

## Methods

### Search Strategy

With the support of an informationist, we searched five databases in January 2021: PubMed, Embase, CINAHL, Web of Science, and PsycINFO. Racism-related search terms included discriminat*, prejudic*, bias, segregat*, and racis*. Aging-related search terms included: aged, older, elder*, senior, and geriatric*. To capture studies that included Black participants, we used the terms Black, Blacks, and African American. We used the truncation operator (*) to capture all words that contain a particular root (e.g., discriminat* for discriminate, discrimination, discriminated, discriminating, discriminatory, discriminates) in databases that allow. In databases that did not allow, we wrote out the multiple words sharing a trunk as separate search terms.

### Selection Criteria

We included articles describing peer-reviewed, empirical, quantitative studies based in the USA that used a measure of racism as an independent variable and a health outcome as a dependent variable, included only participants over the age of 50 years or specifically reported results for participants over the age of 50, and included only Black participants or specifically reported results for Black participants. We selected 50 years as the age cutoff in this study as opposed to a later one out of consideration that in 2018, non-Hispanic Black male populations still presented with the lowest life expectancy at birth (71.3 years as compared to non-Hispanic White males, 76.2 years; non-Hispanic Black females, 78.0 years; and non-Hispanic White females, 81.1 years) [19]. We excluded studies published more than 10 years ago due to the clarification of concepts over the past decade, such as the levels of racism. [5] We included studies that identified “racial composition” or a related term as an independent variable if the paper identified “racism” or “discrimination” as a concept of interest in the abstract, introduction, and/or methods of the manuscript.

### Study Identification

**Fig. 1:**
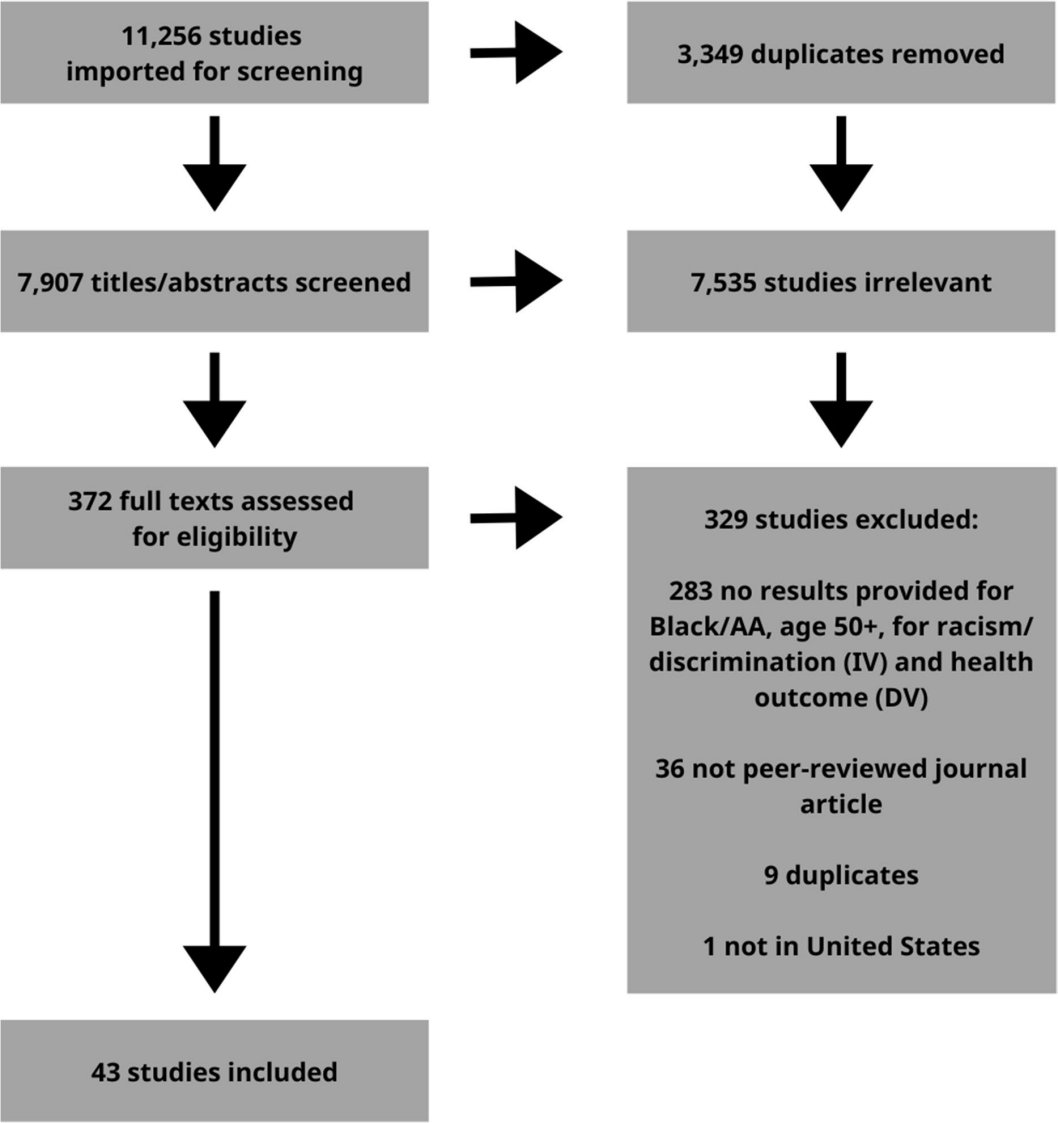
Article selection process (PRISMA diagram)

We identified 11,256 articles for screening and imported them into Covidence software for review. [20] After removing 3349 duplicates, 7907 studies remained for title and abstract screening. Two study team members (SEL and QS) each reviewed all study titles and abstracts to remove irrelevant studies. A third study team member was available to resolve conflicts but was not requested to do so since both study team members came to consensus through discussions and rereviewing abstracts. During the title and abstract screening, we removed 7535 references due to irrelevance. A total of 372 studies remained for full-text screening which one study team member (SEL) completed. Out of the total studies, we excluded an additional 329 manuscripts due to not providing results on the direct relationship between racism and one or more health outcomes among Black adults aged 50 or older (283), not being peer-reviewed (36), being further duplicates of included studies (9), and not studying participants in the USA (1). After removing these 329 studies, 43 studies remained for data extraction. [21–62] Two study team members (SEL and either JJS, AB, or MCF) independently completed data extraction for each included article and cross-checked all results to ensure the accuracy of extraction. See Fig. 1 for the PRISMA diagram.

### Data Extraction

For each of the 43 included studies, we recorded characteristics including racial/ethnic groups included in sample, inclusion and exclusion criteria, parent study or dataset, measure(s) of racism and if validated, measure(s) of health, study design, sample size, and association(s) between racism and health. For a racism instrument to be considered validated, authors needed to provide validation statistics, provide a citation for a validity/reliability study of the instrument, or use an objective data source (e.g., statistics of racial composition in a census tract). If a study measured a health outcome as well as other dependent variables such as a measure of treatment type or patient experience, we only extracted the findings related to health outcomes. If a study included younger participants or non-Black participants, we only extracted the findings in Black participants aged 50 years or older.

We categorized health outcome measures as “cardiovascular,” “mental health,” “cognition,” “physical function,” “telomere length,” or “general health/other.” We categorized racism measures as “perceived interpersonal,” “perceived institutional,” “institutional indicator” (assessing exposure to a racist policy in one context, such as education, based on participant zip code) or “structural indicators.” We only classified measures of racism that assessed exposure to policies, practices, systems, or structures across two or more contexts (e.g., education and employment) as assessing at the structural level of racism. Although there is lack of consensus on the distinction between institutional and structural racism, leading scholars support this conceptualization of structural racism. [5, 63]

See Table 1 for a detailed summary of each included study and Table 2 for a quick reference of study characteristics and findings.

**Table 1:** Summary of the included study

First author, year	Sample size and ages*	Racism measure(s)	Health outcome(s) measure(s)	Findings in older Black adults (significant at *p* < 0.05 unless otherwise specified)	Study design
Aiken-Morgan 2015	420, ages 50 +	School segregation (self-report)	Change in cognition over a 3-year period; tests of global cognition, reasoning, memory, working memory, language, perceptual speed	The desegregated group scored better for language (desegregated: 52.438 ± 0.720 vs. segregated: 49.404 ± 0.577; *F*(1412) = 10.586, *p* = 0.001) and perceptual speed (desegregated: 52.970 ± 0.651 vs. segregated: 50.461 ± 0.521; *F*(1412) = 8.856, *p* = 0.003)	Longitudinal
Assari 2016	3648 aggregated total, sample sizes for older Black adult strata are unspecified (middle age: 35–65; older aged: 66–89; Black and White)	"Everyday discrimination" and "lifetime discrimination"; author doesn't cite or name Williams but description of measures suggests that the Everyday Discrimination and Major Experiences of Discrimination Scale may have been used	Body mass index	None	Cross-sectional
Ayalon 2011	956 (everyday discrimination) and 950 (major lifetime discrimination), ages 50 +	Everyday Discrimination (Williams) and Major Experiences of Discrimination	Depression (CES-D)	Increased major lifetime discrimination and everyday discrimination associated with depressive symptoms; (*β* = 0.16, SE 0.04, *p* < 0.001) and (*β* = 0.16, SE 0.05, *p* < 0.01), respectively	Cross-sectional
Barnes 2012	407, ages 65 +	Everyday Discrimination (Williams)	Tests of cognition in episodic memory, semantic memory, working memory, perceptual speed, visuospatial ability	Perceived discrimination is negatively associated with global cognition (*β* = − 0.02; SE = 0.01), and with two of the five domains: episodic memory, *β* = − 0.03; SE = 0.01 and perceptual speed, *β* = − 0.04; SE = 0.02	Cross-sectional
Beatty Moody 2019	23, ages 54.41–69.40	1 item from Major Experiences of Discrimination (Williams); Experiences of Discrimination Scale (Krieger)	White matter lesion volume (WMLV) using structural MRI	As major experiences burden increased, WMLV increased (*β* = 0.65, *p* = 0.016); as experiences of discrimination increased, WMLV increased (*β* = 0.22, *p* = 0.024)	Cross-sectional
Bor 2018	34,612, ages 50–64; 28,973, ages 65 +	Number of police killings of unarmed Black Americans occurring 3 months prior to interview in the participant’s state of residence	Self-reported mental health (number of days reported “not good” in the previous month)	In participants aged 50–64, number of police killings of unarmed Black Americans in 3 months prior associated with an increase in poor mental health (*β* = 0.27, 95% CI: 0.057, 0.48, *p* = 0.014); not significant in those aged 65 +	Cross-sectional
Chae 2012	1490, ages 50 +	Major Experiences of Discrimination and Everyday Discrimination (Williams)	Mood disorder (major depression, dysthymia, bipolar disorder) (World Mental Health Organization Composite International Diagnostic Interview (WMH-CIDI)); cardiovascular disease history (self-report of hypertension, atherosclerosis, heart attack, stroke)	None	Cross-sectional
Clay 2015	251, ages 65 +	Single question about experiences of discrimination based on race or skin color in past 6 months	Short physical performance battery (SPPB)	None	Cross-sectional
Cobb 2020	964, ages 50 +	Major Experiences of Discrimination (Williams)	C-reactive protein (inflammatory biomarker)	Major discrimination associated with high-risk CRP (PR: 1.36, 95% CI: 1.25–1.47)	Cross-sectional
Cole 2017	1139, ages 50 +	Residential segregation (census tract)	Stage of hypertension (average of 3 readings)	None	Cross-sectional
Coley 2017	5652, ages 65 +	Everyday Discrimination (Williams)	Health-related quality of life (HRQOL) (2 NHANES questions)	Higher perceived discrimination associated with worse overall HRQOL (OR = 1.11; 95% CI: 1.08, 1.15), with stronger effects for women in overall and mental HRQOL	Cross-sectional
Greer 2014	265 metropolitan statistical areas (MSAs) with 935 counties total; minimum of 5000 non-Hispanic Black residents per MSA, ages 65 +	Residential segregation (census tract)	Heart disease and stroke mortality	Segregation positively associated with heart disease mortality rates (RR = 1.13; 95% CI: 1.08, 1.19) in people aged 65 +	Cross-sectional
Han 2020	124, ages 65 +	Everyday Discrimination (Williams)	Tests of cognition in memory, semantic memory, visuospatial ability, perceptual speed, working memory; 3 T MRI brain scan to assess functional connectivity	Discrimination associated with stronger functional connectivity between the left insula and bilateral intracalcarine cortex, weaker functional connectivity between the left insula and right dorsolateral prefrontal cortex (cluster size = 471 voxels, *t*-value = − 4.91, FDR cluster *p* = 0.016), and weaker functional connectivity between the right insula and left supplementary motor cortex (cluster size = 778 voxels, *t*-value = − 5.26, FDR cluster *p* = 0.002)	Cross-sectional
Kim 2017	429, ages 55 +	Everyday Discrimination (Williams)	Past-year psychiatric disorder (World Health Organization Composite International Diagnostic Interview-CIDI)	Greater perceived discrimination associated with increased odds of having any past-year psychiatric disorder with results varying by region (stronger in the West (odds ratio [OR] = 1.44, 95% CI: 1.12, 1.85) than in the South (OR = 1.06, 95% CI: 1.01, 1.11))	Cross-sectional
Kovalchik 2015	437, ages 50 +	Racial composition (census tract) and residential segregation (county)	Global cognitive functioning (Telephone Interview for Cognitive Status-TICS)	None	Longitudinal
Krieger 2014	National mortality data, deaths before age 65 years	State did or did not have legal racial discrimination overturned by 1964 Civil Rights Act	Premature mortality (< 65 years old)	A temporal pattern emerged for Black people across the twentieth century (higher Jim Crow–related mortality for oldest group, followed by no difference, then smaller reemerging difference)	Cross-sectional
Krieger 2017	43,384, ages 52 +	Birth in a Jim Crow State	Estrogen-receptor (ER)-negative breast tumors	Odds of ER − versus ER + cancer for those born in Jim Crow state: 1.10 (95% CI: 1.01, 1.18) for those born in or before 1945 and 1.10 (95% CI: 1.02, 1.20) for those born in 1946–1965	Cross-sectional
Lamar 2020	497, ages 65 +	Region of birth and residence and school segregation status (self-reported)	Tests of global cognition episodic memory, semantic memory, working memory, perceptual speed, and visuospatial ability	Southern birth predicted lower global cognitive functioning (estimate = − 0.22, SE = 0.04, *p* < 0.0001), lower levels of performance in episodic memory, semantic memory, working memory, perceptual speed, and visuospatial ability; southern birth did not predict change in cognition over time. Southern residence at age 12 associated with lower level of global cognitive functioning (estimate = − 0.20, SE = 0.04, *p* < 0.0001), and all of the cognitive domains except episodic memory, but not change over time; school segregation status not associated with either baseline levels or rates of change in any of the cognitive outcomes (*p*-values ≥ 0.08)	Longitudinal
Lee 2017	595, ages 50 +	Major Experiences of Discrimination (Williams)	Leukocyte telomere length	Increased experiences of discrimination were associated with shorter telomere length (age-adjusted: *β* = − 0.033, SE = 0.14, *p* = 0.018; sociodemographic factors-adjusted: *β* = − 0.034, SE = 0.14, *p* = 0.017; health-related factors-adjusted: *β* = − 0.034, SE = 0.14, *p* = 0.016; depressive symptoms-adjusted: *β* = − 0.034, SE = 0.14, *p* = 0.017; stress-related factors-adjusted: *β* = − 0.030, SE = 0.15, *p* = 0.046)	Cross-sectional
Linnenbringer 2020	2273, ages 65 +	Neighborhood racial composition (census block group)	Breast cancer subtype (ER/PR/HER2 expression)	Black women who were diagnosed with breast cancer at age 65 or older had a 5.8% lower odds of TNBC vs. HR + /HER2 − breast cancer (OR = 0.94; 95% CI: 0.90, 0.99) per 10% unit increase in block group percentage Black (greater concentration of Black neighbors)	Cross-sectional
Liu 2015	1956 aggregated total, sample sizes for older Black strata are unspecified (ages 25–74 during years 1971–75 and 1976–80 of NHANES)	School term length during Jim Crow (disparities in education quality)	Blood pressure and hypertension (readings taken)	Among Black women, a 10% longer school term length was associated with a 2.1 mmHg lower systolic blood pressure (95% CI: − 4.1, − 0.1), 1.0 mmHg lower diastolic blood pressure (95% CI: − 2.2, − 0.1), and 5.0 percentage points lower hypertension prevalence (95% CI: − 8.4, − 1.7) in adjusted models. Associations for Black men were not statistically significant	Cross-sectional
Liu 2017	550, ages 50+	Major Experiences of Discrimination and Everyday Discrimination (Williams)	Leukocyte telomere length	Everyday discrimination, but not major discrimination, is associated with shorter leukocyte telomere length among Black older adults (*β* = –0.23; 95% CI: –0.44, 0.01) but not among white counterparts (*β* = 0.05; 95% CI: –0.01, 0.10)	Cross-sectional
Lu 2019	339, ages 60 +	Major Experiences of Discrimination and Everyday Discrimination (Williams)	Relative telomere length	None	Cross-sectional
Marshall-Fabien 2016**	1108, ages 55 +	Everyday Discrimination (Williams)	Depression (CES-D)	Discrimination is associated with depression for overall sample (*β* = 1.42, *p* < 0.001) and among African American stratum (*β* = 1.41, *p* < 0.001); association among stratum with English-speaking Black participants from the Caribbean was not significant	Cross-sectional
Marshall 2012**	1108, ages 55 +	Everyday Discrimination (Williams)	Depression (CES-D)	Discrimination associated with depression (*β* = 1.52, 0.29 SE, *p* < 0.001 in total sample)	Cross-sectional
McClendon 2019	289, ages 60.7–73.3	Major Experiences of Discrimination (Williams)	Health-related quality of life (RAND SF36)	Structural equation modeling showed race had an indirect effect through discrimination and various personality traits on physical health (neuroticism: − 0.03, 95% CI: − 0.04, -0.02; conscientiousness: − 0.13, 95% CI: − 0.20, − 0.06), and mental health (neuroticism: − 0.05; 95% CI: − 0.07, − 0.03; agreeableness: − 0.01, 95% CI: − 0.02, − 0.00)	Cross-sectional
Mezuk 2011	445, ages 50 +	Workplace Discrimination (Williams)	Hypertension (based on readings or self-reporting taking anti-hypertensives)	None	Cross-sectional
Mouzon 2017	773, ages 55–93	Everyday Discrimination (Williams)	Lifetime mood disorders; lifetime anxiety disorders; depressive symptoms (CES-D); and serious psychological distress (Kessler 6)	Higher levels of overall everyday discrimination was associated with higher odds of mood disorder (1.05, 95% CI: 1.02, 1.08, *p* = 0.003), anxiety disorder (1.05, 95% CI: 1.02, 1.08, *p* = 0.003), any disorder (1.06, 95% CI: 1.04, 1.09, *p* < 0.0001), number of lifetime DSM-IV disorders (1.03, 95% CI: 1.02, 1.04, *p* < 0.0001), elevated levels of depressive symptoms (1.02, 95% CI: 1.01, 1.03, *p* < 0.0001), and serious psychological distress (1.03, 95% CI: 1.02, 1.05, *p* < 0.0001)	Cross-sectional
Nadimpalli 2015	487, ages 60–98	Everyday Discrimination (Williams)	Depression (CES-D)	Perceived discrimination was positively associated with depressive symptoms (OR: 1.20, 95% CI: 1.10, 1.31; *p* < .001)	Cross-sectional
Nguyen 2018	278, ages 55 +	Everyday Discrimination (Williams)	Serious Psychological Distress (Kessler 6)	Discrimination associated with serious psychological distress (0.03 (0.01), *p* < 0.05)	Cross-sectional
Nguyen 2019	3742 observations (some participants included at more than one observation), ages 50 +	One item from the Everyday Discrimination Scale (Williams) specific to healthcare	Biomarkers of cardiometabolic risk: high sensitivity C-reactive protein (CRP), hemoglobin A1c (HbA1c), high-density lipoprotein (HDL), total cholesterol, cystatin C and blood pressure	Those who reported discrimination in the health care setting had increased likelihood of elevated CRP (OR 1.55, CI 1.34, 1.79; *p* < 0.001), elevated HbA1c (OR 2.03, CI 1.70, 2.41; *p* < 0.001) and elevated blood pressure (OR 1.64, CI 1.45, 1.86; *p* < 0.001), decreased likelihood of low HDL (OR 0.86, CI 0.74, 1.00; *p* < 0.05), decreased likelihood of high total cholesterol (OR 0.82, CI 0.67, 1.00; *p* < 0.05)	Cross-sectional
Nkimbeng 2020	165, ages 55 +	Everyday Discrimination (Williams)	Physical function (PROMIS PF 10a)	High discrimination associated with 2.5 points lower physical functioning compared to low discrimination (*β* = − 2.51, 95% CI: − 4.84, − 0.17)	Cross-sectional
Pantesco 2018	176 aggregated total, sample sizes for older Black strata are unspecified (ages 30–64)	Ten items about experiences of prejudice or discrimination (Laveist); two items from Major Experiences Scale (Williams); 5 items from Experiences of Discrimination Scale (Krieger); Everyday Discrimination Scale (Williams)	Telomere length	None	Cross-sectional
Pugh 2021	617, ages 57 +	Everyday Discrimination (Williams)	Tests of global cognition, episodic memory, working memory, semantic memory, perceptual orientation, perceptual speed	Discrimination associated with better performance in semantic memory over time and with better working memory	Longitudinal
Taylor 2018	120, ages 50–80	General Ethnic Discrimination Scale (GED) adapted from Schedule of Racist Events Scale	Pain intensity (McGill Pain Questionnaire—SFMPQ); depression (CES-D)	Racial discrimination associated with pain intensity (*β* = 9.45) and depression (*β* = .71); pain intensity no longer significant after adding depression to the models and a mediation effect was detected	Cross-sectional
Vásquez 2019	1960, ages 55 +	Study-specific measure of perceived interpersonal and perceived institutional discrimination across 6 domains	Body mass index	None	Cross-sectional
Walker 2016	120, ages 50–80	General Ethnic Discrimination Scale adapted from Schedule of Racist Events Scale	Functional limitations (Health Assessment Questionnaire Disability Index—HAQ-DI); disability (Craig Handicap Assessment and Reporting Technique—CHART); depression (CES-D)	Racial discrimination associated with disability (*β* = − 132.46) and depression (*β* = 0.65)	Cross-sectional
Watkins 2011	300, ages 55 +	Everyday Discrimination (Williams)	Depression (CES-D)	None	Cross-sectional
Wheaton 2018	92, ages 55 +	Major Experiences of Discrimination and Everyday Discrimination (Williams)	Depression (CES-D)	Among older men, only major discrimination predicted elevated symptoms of depression with both forms of discrimination considered (compared to low major discrimination, moderate: 5.20, SE 1.94 and high: 4.54, SE 1.86); both major and everyday discrimination were associated with the depressive symptoms in older men when considered individually (specific results not provided)	Cross-sectional
White 2011	689, ages 65 +	Residential segregation (Wong’s local index modeling potential for interaction between Blacks and non-Blacks)	Hypertension (self-report of diagnosis)	Foreign-born Blacks aged 65 or older residing in highly segregated areas were 46% (PR: 0.54; 95% CI: 0.40, 0.72) less likely to report hypertension than their counterparts residing in low segregated areas; no significant association found for US-born Blacks	Cross-sectional
White 2020	2926, ages 50 +	Everyday Discrimination (Williams)	Depression (CES-D)	Respondents in the persistently high racial discrimination trajectory were associated with elevated depressive symptoms (IRR: 1.50; 95% CI: 1.29, 1.73) in comparison to respondents in low to moderate perceived racial discriminatory trajectory	Longitudinal
Yoon 2019	397, ages 65 +	Major Experiences of Discrimination and Everyday Discrimination (Williams)	Self-reported mental health (mental component summary (MCS) of SF36); self-report of mental health not good after thinking about their mental health) stress, depression, and problems with emotions) in the past 30 days; self-report of diagnosed anxiety/depression	Everyday discrimination associated with worse mental health in men (*β* = − 0.197) and women (*β* = − 0.304, *p* < .001); Major Experiences of Discrimination did not predict mental health outcomes for either gender	Cross-sectional
Zahodne 2019	1313, ages 65 +	Everyday Discrimination (Williams)	Episodic memory via a telephone assessment	Structural equation modeling showed greater perceived discrimination had a direct effect on faster memory decline (estimate: -0.010, SE: 0.005, *p* = 0.048) and lower initial memory (estimate: 0.003, SE: 0.002, *p* = 0.053) via depressive symptoms and external locus of control	Longitudinal

^*^If a study included younger participants or participants of a race besides Black, sample size is given for the older Black strata unless otherwise specified

^**^Same study sample (were looking at different moderators/interactions)

**Table 2:** Study characteristics and findings

First author, year	Sample size				Racial/ethnic groups included in addition to Black			Discrimination measured				Health categories associated with racism in older Black adults							Validated discrimination measure		Study design	
	< 100	100–1000	1000–5000	5000 +	White	Hispanic/ Latinx	None	Perceived interpersonal	Perceived institutional	Institutional indicator	Structural indicators	Cardiovascular	Mental health	Cognitive	General health/other	Physical function	Telomere length	None	Yes*	No	Cross-sectional	Longitudinal
Aiken-Morgan 2015		X					X			X				X						X		X
Assari 2016			X**		X			X	X									X		X	X	
Ayalon 2011			X		X	X		X	X				X						X		X	
Barnes 2012		X					X	X						X					X		X	
Beatty Moody 2019	X						X		X					X					X		X	
Bor 2018				X	X					X			X						X		X	
Chae 2012			X				X	X	X									X	X		X	
Clay 2015		X			X			X										X		X	X	
Cobb 2020		X			X	X			X			X							X		X	
Cole 2017			X				X			X								X	X		X	
Coley 2017				X			X	X							X				X		X	
Greer 2014				X	X					X		X							X		X	
Han 2020		X					X	X						X <					X		X	
Kim 2017		X					X	X					X						X		X	
Kovalchik 2015		X			X	X				X								X	X			X
Krieger 2014				X	X						X				X				X		X	
Krieger 2017				X	X						X				X				X		X	
Lamar 2020		X					X				X			X					X	X		X
Lee 2017		X					X		X								X		X		X	
Linnenbringer 2020			X		X					X					X				X		X	
Liu 2015			X		X					X		X							X		X	
Liu 2017			X		X			X	X								X		X		X	
Lu 2019		X					X	X	X									X	X		X	
Marshall-Fabien 2016 !			X				X	X					X						X		X	
Marshall 2012 !			X				X	X					X						X		X	
McClendon 2019		X			X				X				X			X			X		X	
Mezuk 2011		X			X	X			X									X	X		X	
Mouzon 2017		X					X	X					X						X		X	
Nadimpalli 2015		X					X	X					X						X		X	
Nguyen 2018		X					X	X					X						X		X	
Nguyen 2019			X		X	X		X				X/and X <								X ~	X	
Nkimbeng 2020		X					X	X								X			X		X	
Pantesco 2018		X			X			X	X								X		X		X	
Pugh 2021		X					X	X						X <					X			X
Taylor 2018		X!					X	X	X				X		X				X		X	
Vásquez 2019			X		X	X		X	X									X		X	X	
Walker 2016		X!					X	X	X				X			X			X		X	
Watkins 2011		X					X	X										X	X		X	
Wheaton 2018	X						X	X	X				X						X		X	
White 2011			X				X			X		X <								X	X	
White 2020			X				X	X					X						X			X
Yoon 2019		X					X	X	X				X						X		X	
Zahodne 2019			X		X	X		X						X					X			X
Number of studies (of 43)	2	22	14	5	18	7	26	27	16	8	3	5	14	7	5	2	3	10	37	7	37	6

^*^Validity/reliability information provided in the manuscript, a citation for a validity/reliability study of the measurement is provided, or a simple data source (e.g., state of birth) is used

!Same study samples

< Racism associated with positive health outcome (opposite direction from hypothesis)

~ Study used a modified version of a validated tool and did not provide validity/reliability statistics for a modified version

^**^Sample size for study; strata size unspecified for older Black adults

## Results

### Study Characteristics

Of the 43 included studies, 13 were published in health outcome-specific journals [25, 33, 35, 37, 38, 42, 44, 47, 51, 56, 58, 59, 62] (e.g., *Breast Cancer Research and Treatment*), 15 in aging-specific journals [21, 22, 24, 28, 30, 32, 34, 36, 45, 53–55, 57, 60, 61] (e.g., *Journals of Gerontology*), four in racial disparities or race-specific journal [23, 41, 49, 52] (e.g., *Journal of Racial and Ethnic Health Disparities*) and the remainder in general health and social science journals [26, 27, 29, 31, 39, 40, 43, 46, 48, 50, 64] (e.g., *PLOS One*).

Twenty-six of the studies included only Black participants, [21, 23, 24, 27, 28, 31–34, 37, 38, 40, 42, 44, 45, 49, 50, 53–55, 57–62] 18 included Black and White participants only, [22, 25, 26, 29, 30, 35, 36, 39, 41, 43, 46–48, 51, 52, 56, 61, 64] and seven included Black, White, and Hispanic (of any race) participants. [22, 29, 30, 36, 46, 52, 61] None of the eligible studies included other racial or ethnic groups (e.g., American Indian, Asian).

### Health Outcome Variables

Cardiovascular measures included cardiometabolic biomarkers such as C-reactive protein (CRP), [22, 29] hypertension/blood pressure, [23, 29, 33, 52, 64] and having either a history of or dying from heart disease. [40, 43] Cognitive measures included single and multi-component tests of cognition [21, 24, 37, 44, 46, 57, 61] and neuroimaging assessments. [38, 44] Physical function measures included questionnaires and battery assessments of function, disability, and physical performance. 33,44,48,52 Mental health measures included measured and self-reported depression, [28, 31, 32, 34, 36, 49, 50, 53, 59, 60] self-reported mental health quality, [34, 39, 51] measured and self-reported mood, psychiatric or anxiety disorder, [34, 40, 45, 53] and measured psychological distress. [53, 54] Four studies exclusively assessed telomere length. [26, 27, 56, 62] General/other health measures included body mass index, [30, 35] health-related quality of life, [42, 51] premature mortality, [48] pain intensity, [58] and breast cancer subtype. [25, 47]

### Racism Variables

Twenty-seven studies included measures of perceived interpersonal racism either alone or in combination with perceived institutional measures. Measures of perceived interpersonal racism included either a portion of or the entire Williams Everyday Discrimination Scale, [26–29, 31, 32, 34–37, 40, 42, 44, 45, 49, 50, 53–57, 60, 61] the Schedule of Racist Events (a portion of which assesses interpersonal experiences) and the General Ethnic Discrimination Scale adapted from it, [58, 59] and questions written either by the study authors or other cited authors about perceived interpersonal racism. [30, 41, 56]

Sixteen studies included measures of perceived institutional racism either alone or in combination with perceived interpersonal measures. Measures of perceived institutional racism included the Williams Major Experiences of Discrimination Scale, [22, 26, 27, 32, 34–36, 38, 40, 51, 56, 62] the Krieger Experiences of Discrimination Scale, [38, 56] the Schedule of Racist Events (a portion of which assesses institutional experiences) and the General Ethnic Discrimination Scale adapted from it, [58, 59] the Williams Workplace Discrimination Scale, [52] and questions written by the study authors about perceived institutional racism. [30]

Eight studies used indicators of racist policies or practices within one context to assess participant exposure to institutional racism. Institutional indicators included residential segregation (e.g., isolation index), [23, 25, 33, 43, 46] school segregation, [21, 24] school term length, [64] and number of recent police killings of unarmed Black Americans in the participant’s state of residence. [39]

Three studies assessed exposure to structural racism. All three measured whether a person was born/had resided in a Jim Crow state or region. [24, 47, 48] This was categorized as “structural” because Jim Crow laws affected a person across contexts (e.g., attending school, buying a home). One of these studies also assessed school segregation. [24]

### Health Outcomes

Thirty-three of the 43 studies found a significant association between racism and health in Black adults aged 50 or older. Of these, 14 found significant associations between racism and mental health, [28, 32, 34, 36, 39, 45, 49–51, 53, 54, 58–60] seven for cognition, [21, 24, 37, 38, 44, 57, 61] five for cardiovascular outcomes, [22, 29, 33, 43, 64] three for physical function, [51, 55, 59] two for telomere length, [26, 62] and five for general/other health outcomes. [25, 42, 47, 48, 54] Specific significant findings are included below based on health category of dependent variable(s) and level of racism of independent variable(s). Unless specified otherwise, only statistically significant results found for older Black adults are included in the sections below.

### Associations Between Racism and Mental Health

In studies that assessed mental health, interpersonal measures were significantly and positively associated with depressive symptoms, [32, 36, 49, 50, 53, 58–60] psychological distress, [53, 54] worse self-reported mental health, [34] psychiatric disorders, [45, 53] mood disorders, [53] and anxiety. [53] For example, one study found that African American participants aged 60 years and older who reported experiencing discrimination “often” or “sometimes” on the Everyday Discrimination Scale were 20% more likely to report depressive symptoms on the CES-D after controlling for age, sex, education and income, as compared to those who reported experiencing discrimination “rarely” or “never.” [28] In another study, higher levels of everyday discrimination (measured continuously) were associated with worse mental health as reported on the SF36 Mental Health Component Summary. [34]

Five studies found significant associations between perceived institutional racism and mental health outcomes. A study that assessed the relationship between perceived institutional discrimination and health-related quality of life found that increased discrimination was associated with poorer mental health, one component of the quality of life scale. [51] Three studies that used measures of both interpersonal and institutional racism found an association with depression. [36, 58, 59]

One study assessed the relationship between an indicator of institutional racism and self-reported mental health. In that study, the number of police killings of unarmed Black Americans in the past three months was associated with an increase in poor mental health among participants aged 50–64 years (but not among those aged 65 and older). [39]

### Associations Between Racism and Cardiovascular Health

One study found significant associations between interpersonal discrimination and cardiovascular health: Non-Hispanic Black participants who reported discrimination in the health care setting, using one item from the Everyday Discrimination Scale, had an increased likelihood of elevated CRP, elevated HbA1c, and elevated blood pressure. [29] Contrary to hypotheses, Black participants who reported discrimination in this study had lower levels of total cholesterol and lower levels of HDL compared to Black participants who did not report discrimination. [29]

One study found an association between perceived institutional discrimination and elevated CRP, an inflammatory biomarker linked with experiences of heightened stress. [22] In another, a 10% longer school term length was associated with lower systolic blood pressure, lower diastolic blood pressure, and lower risk of hypertension among Black women; there was no association in Black men. [64] Shorter term length is an indicator of discrimination against quality education particularly during the Jim Crow Era. In a third study using institutional indicators, researchers used a novel measure of residential segregation: Wong’s Local Index models the potential for interaction between people of the same and of different races. [33] Foreign-born Black participants aged 65 or older were 46% less likely to report hypertension if they lived in high-segregation areas than if they lived in low-segregation areas. Researchers did not find a significant association among US-born Black participants. [33] Conversely, a different study found that racial residential segregation was associated with heart disease mortality among Black people aged 65 and older. [43]

### Associations Between Racism and Cognitive Health

Three studies found a significant association between everyday discrimination and poorer performance on one or more tests of cognition. [37, 57, 61] In addition, attending a segregated school as a child was associated with poorer language and perceptual speed scores in a battery of cognitive tests. [21] In this longitudinal study, there were no significant associations between school segregation and change in cognition over time or between school segregation and baseline global cognition, reasoning, or memory. [21] In another study, birth in the southern US predicted lower cognitive functioning, including lower levels of performance in episodic memory, semantic memory, working memory, perceptual speed and visuospatial ability. [24] Southern US residence at age 12 years was associated with lower levels of global cognitive functioning and all cognitive domains except episodic memory. [24] Neither southern US residence at age 12 years nor birth in the southern US predicted change in cognition over time. School segregation status was not associated with either baseline levels or rates of change in any of the cognitive measures. [24]

Two studies assessed the relationship between perceived interpersonal or institutional discrimination and changes in brain imaging. One found that everyday discrimination was associated with stronger functional connectivity between the left insula and bilateral intracalcarine cortex, weaker functional connectivity between the left insula and right dorsolateral prefrontal cortex, and weaker functional connectivity between the right insula and left supplementary motor cortex. [44] Another found that perceived institutional discrimination was associated with increased white matter legion volume. [38]

### Associations Between Racism and Telomere Length

Of the four studies that assessed discrimination and telomere length, two found significant associations between discrimination and shorter telomere length (an indicator of biological aging). In one of the studies, major discrimination was associated with shorter telomere length among African American males and females aged 51 years and older after controlling for sociodemographic and health factors such as depressive symptoms and stress. [62] In the other, everyday discrimination was associated with shorter telomere length among non-Hispanic Black participants aged 50 years and older, though this association was not observed with experiences of major discrimination. [26]

### Associations Between Racism and Physical Function

Three studies found an association between racism and physical function. In one, higher scores on a scale measuring both perceived interpersonal and perceived institutional racism were associated with disability, using functional limitations and disability questionnaires. [59] In another, perceived everyday discrimination was associated with worse physical functioning using the PROMIS physical functioning measure. [55] In the third, perceived institutional discrimination was associated with poor self-reported health. [51]

### Associations Between Racism and General/Other Health Outcomes

We classified associations between racism and outcomes as “general/other health” if they were not otherwise categorized. One study found that neighborhood segregation was associated with decreased likelihood of high-risk breast cancer subtype, contrary to the hypothesis. [25] Another found that everyday discrimination was associated with worse overall health-related quality of life. [42] In a study using a measure of both perceived interpersonal and perceived institutional discrimination, discrimination was associated with greater pain intensity; this relationship was no longer significant after adding depression to the model and a mediation effect was detected. [58]

Two studies by the same first author assessed the relationship between birth in a Jim Crow state (an indicator of exposure to structural racism) and health outcomes. One study found that the odds of having more aggressive subtypes of cancer (ER − versus ER +) were higher for those born in a Jim Crow state. [47] The other found that residence in a Jim Crow state was associated with premature mortality for older Black Americans, and the relationship was strongest for those who were oldest during the Jim Crow Era (i.e., had greater degrees of exposure to Jim Crow policies over time). [48]

### Study Design

While 37 of the studies were cross-sectional in design, six were longitudinal. [21, 24, 46, 57, 60, 61] As indicated in Table 2, sample sizes for older Black American participants across all 43 included studies were categorized as small (*n* =  < 100), medium (*n* = 100–1000), large (*n* = 1000–5000), and very large (*n* = 5000 +). Very large studies typically used census-tract data or other area-level variables. Many of the studies used data from the same large parent studies such as the Health and Retirement Study [22, 26, 29, 36, 46, 52, 60–62] and the Minority Aging Research Study. [28, 37, 44, 57]

Thirty-six of the studies used validated measures of discrimination and six [21, 29, 30, 33, 35, 41] did not (or did not provide evidence of psychometrics validation). One study used both a validated and an unvalidated measure. [24] Unvalidated measures included self-report of school segregation, [21, 24] a single question taken from a validated instrument (no validity or reliability statistics were provided), [29] a study-specific measure of perceived interpersonal and institutional racism (no validity or reliability statistics were provided), [30] a single question written by the study authors (no validity or reliability statistics were provided), [41] and a novel measure of racial segregation (no validity or reliability statistics were provided or could be found in the cited references). [33] Another study’s authors used terminology similar to the Williams Everyday and Major Experiences scales (both of which are widely validated [65]) when describing discrimination measurement, but did not name the scales used, cite them, or provide validity statistics; it is possible that this study used the validated scales. [35]

See Tables 1 and 2 for detailed study characteristics and results.

## Discussion

### Racism and Older Adult Health

The findings from this systematic review suggest that there are important associations between experiences of racism and older Black American adult health across categories of health outcomes and levels of racism. While there is a large and growing body of evidence regarding the relationship between racism and health outcomes, this review reveals a need for increased focus on this area in older adults specifically. Past reviews have found hundreds of articles examining racism and health in general populations, [11, 16] while this review identified just 43 articles that reported results exclusively or specifically in Black individuals aged 50 years and older. Considering the multi-level and long-term experiences with racism of many older adults, researchers should consider designing studies that exclusively sample an older adult population or specifically report results of the effects of racism on older adults within a broader population. Some of the results provided in this paper were found in online-only appendices; in studies of mixed-age populations, providing age-stratified results in supplemental materials could be a strategy for providing this important detail while staying within word limits set by publishers.

It is worth emphasizing that research focusing on “older adults” can include people from various age ranges beyond just 50 to 100 + years. However, researchers also look at no other life periods of the age spectrum with as broad of a reference point as they often do with older adults. It is important for researchers in future studies to examine the multilevel differences in exposure to racism and the effects that they have on health both within and between age cohorts of older adults.

Many older Americans today were born before the Jim Crow laws that formalized discriminatory policies and practices based on race were abolished and were also alive when the Social Security Act of 1935 enacted a social retirement benefit that systematically disadvantaged aging Black Americans. [5] All older adults today have witnessed and/or been directly impacted by contemporary racist policies and practices such as the disproportionate incarceration of Black Americans. [9] It is well established that social determinants of health such as the lack of access to resources (e.g., education and job opportunities), negative interpersonal experiences, living in disadvantaged neighborhoods, and other differences across opportunities adversely impact health. The studies in this review suggest that exposures to racism across the life course may be a root cause of many health disparities among older Black Americans.

While there is a need to increase the literature base on older adults and racism in general, there is a particular need for future studies to examine the impacts of racism on older adults across a wide variety of health outcomes and beyond self-reported measures. For example, just three of the studies we reviewed assessed physical function or a related construct (frailty, disability) [41, 55, 59] and only one of those administered an in-person physical assessment. [41] Frailty is an important predictor of healthcare spending and decline among older adults, [66] and its relationship with discrimination should be further—and more rigorously—evaluated.

### Structural Measures

This review revealed an urgent need for the examination of the impact of structural racism in particular on older adult health. The lack of evidence in this area may be related to the lack of consensus regarding the delineation between levels of racism. For example, many researchers use the terms “institutional racism” and “structural racism” interchangeably whereas others, such as Bailey and colleagues, [5] assert that they are distinct concepts. Additionally, there are no widely tested and validated tools that comprehensively measure structural racism, making its study more difficult than the study of interpersonal racism. [7]

While several studies included in this review assessed exposure to institutional racism, most relied on self-reported measures of perception. Since assessing one’s own exposure to systemic forces presents many challenges, more attention is ultimately needed for considering objective measures of institutional and structural racism. Additionally, studies that assessed exposure to institutional racism using objective indicators most often used indicators of neighborhood segregation. No studies included in this review assessed exposure to racism using objective data sources in the contexts of civic life (e.g., voting wait times, government representation), employment, healthcare, media and marketing, environment, or income/credit/wealth. Studies of institutional racism should consider measuring within these contexts, as well as within education, policing, and neighborhood factors. There is a particular need for studies to assess exposures across multiple of these contexts to better measure the downstream effects from structural level racism.

Segregation is often relied upon as a single measure of exposure to racism but this may not be appropriate, particularly for foreign-born individuals. Some studies suggest that a high concentration of immigrants in a neighborhood may have health-protective effects through the promotion of social support enclaves. [21] This is one example that illustrates the importance of measuring across multiple contexts as one metric likely cannot capture a person’s full experiences with racism.

The three studies that did assess exposure to structural racism used state or region of birth (Jim Crow state/region or not). These are valuable contributions to the literature. Additional studies are needed that use institutional indicators from multiple time points in a person’s life (not just historic or just contemporary measures), and at greater geographic specificity (e.g., census tract) to improve understanding the relationship further between cumulative exposures to structural racism and health.

### Multilevel Measures

Results from this review also demonstrate a need for increased use of multilevel measures of racism (i.e., measures across interpersonal, institutional, and structural levels). Eleven of the reviewed studies included measures across more than one level of racism, but all of those used measures of perceived interpersonal and institutional racism; none combined institutional or structural indicators with perceived racism. Theories of racism and discrimination make clear that experiences at one level may impact experiences at another and that there may be synergistic effects; [6] without more rigorous examination of this phenomenon through multi-level measures, the relevance of these theories to an older adult population will remain poorly understood. This issue reinforces the need for better measurement tools that can efficiently and effectively assess racism across contexts (e.g., education, employment, policing), time points, geographic granularities, and levels (i.e., interpersonal, institutional, structural).

### Race of Participants

Another gap in the literature that this search revealed is a need for more racially diverse samples of older adults with analyses based on more nuanced categories. Since race is socially constructed, it may be important to examine differential impacts of racism based on more inclusive categories of race beyond just “Black” and “White.” For example, no studies in this search examined the impacts of variations in skin pigmentation/tone (i.e., effects of colorism) or of perceived versus self-identified race on older adults’ experiences with racism.

Most studies in this search compared Black participants to White participants or included Black participants only. Few studies included multiple minority groups and those that did only provided results specifically for Black participants, Hispanics of any race, and White participants. No studies specifically provided results for participants who identify as Asian American, American Indian, Pacific Islander, or mixed/multiple race. Given this study’s focus, we excluded any studies that did not provide results for Black participants, so while studies that only examined racism in Asian Americans, for example, do exist, [67, 68] they were intentionally excluded. It would be beneficial for future studies to provide results for older adults from other racial groups to enable better understanding of how racial discrimination differentially or similarly affects groups of older Americans.

### Study Design

As in many areas of research, this review revealed that most published studies of racism and older adult health are cross-sectional. Since racism differentially affects cohorts of Americans due to compounding effects from living through multiple iterations of racist policies and cultural attitudes over a lifetime, there is an urgent need for longitudinal studies on this topic in this population.

### Exclusion Based on Incarceration and Institutionalization

Many of the studies in this review specifically excluded individuals who were incarcerated or living in a long-term care setting (e.g., nursing home, group home). Among those that did not specifically exclude based on these criteria, none provided results for these groups specifically or made specific efforts to recruit in these populations. Racism is a particular concern in the criminal justice system and may be an important contributor to an older adult’s inability to age in place due to the impacts of redlining on homeownership and access to community resources. For these reasons, future studies should consider including or focusing on older adults experiencing incarceration or who are living in long-term care facilities.

### Limitations

This review has many strengths, such as the use of a broad and inclusive search strategy. However, there are also some noteworthy limitations. Firstly, though two reviewers completed a title and abstract review to determine the eligibility of a study for inclusion, just one reviewer completed a full-text review to verify eligibility. It is possible that this resulted in some studies being inappropriately excluded. However, specific inclusion and exclusion criteria selected a priori should have mitigated this source of error. While this review supports the need for additional research on institutional and structural racism, a lack of conceptual clarity may also mean that more studies exist that measure racism at the institutional or structural level but that they were not identified in this search because they did not self-identify as such. For example, studies of voting laws that disproportionately impact one race may not use the terms “racism” or “discrimination” in their findings but may in fact be studying these phenomena.

## Conclusion

Researchers in gerontology, public health, and the social sciences should improve measurements of racism and further investigate the relationship between all levels of racism and older Black Americans’ health to improve understanding of how and to what extent racism contributes to racial health disparities.
